# Effects of rapamycin on social interaction deficits and gene expression in mice exposed to valproic acid in utero

**DOI:** 10.1186/s13041-018-0423-2

**Published:** 2019-01-08

**Authors:** Hiroko Kotajima-Murakami, Toshiyuki Kobayashi, Hirofumi Kashii, Atsushi Sato, Yoko Hagino, Miho Tanaka, Yasumasa Nishito, Yukio Takamatsu, Shigeo Uchino, Kazutaka Ikeda

**Affiliations:** 1 Addictive Substance Project, Tokyo Metropolitan Institute of Medical Science, 2-1-6 Kamikitazawa, Setagaya-ku, Tokyo, Japan; 2 Department of Biosciences, School of Science and Engineering, Teikyo University, 1-1 Toyosatodai, Utsunomiya-shi, Tochigi, Japan; 3 Department of Molecular Pathogenesis, Graduate School of Medicine, Juntendo University, 2-1-1 Hongo, Bunkyo-ku, Tokyo, Japan; 4 Department of Pediatrics, Graduate School of Medicine, The University of Tokyo, 7-3-1 Hongo, Bunkyo-ku, Tokyo, Japan; 5 Department of Pediatrics, The University of Tokyo Hospital, 7-3-1 Hongo, Bunkyo-ku, Tokyo, Japan; 6 Department of Developmental Disorders, National Institute of Mental Health, National Center of Neurology and Psychiatry, 4-1-1 Higashimachi, Kodaira-shi, Tokyo, Japan; 7 Center for Basic Technology Research, Tokyo Metropolitan Institute of Medical Science, 2-1-6 Kamikitazawa, Setagaya-ku, Tokyo, Japan

**Keywords:** Autism spectrum disorder, mTOR signaling pathway, Valproic acid, Rapamycin

## Abstract

The mammalian target of rapamycin (mTOR) signaling pathway plays a crucial role in cell metabolism, growth, and proliferation. The overactivation of mTOR has been implicated in the pathogenesis of syndromic autism spectrum disorder (ASD), such as tuberous sclerosis complex (TSC). Treatment with the mTOR inhibitor rapamycin improved social interaction deficits in mouse models of TSC. Prenatal exposure to valproic acid (VPA) increases the incidence of ASD. Rodent pups that are exposed to VPA in utero have been used as an animal model of ASD. Activation of the mTOR signaling pathway was recently observed in rodents that were exposed to VPA in utero, and rapamycin ameliorated social interaction deficits. The present study investigated the effect of rapamycin on social interaction deficits in both adolescence and adulthood, and gene expressions in mice that were exposed to VPA in utero. We subcutaneously injected 600 mg/kg VPA in pregnant mice on gestational day 12.5 and used the pups as a model of ASD. The pups were intraperitoneally injected with rapamycin or an equal volume of vehicle once daily for 2 consecutive days. The social interaction test was conducted in the offspring after the last rapamycin administration at 5–6 weeks of ages (adolescence) or 10–11 weeks of age (adulthood). Whole brains were collected after the social interaction test in the adulthood, and microarray and Western blot analyses were performed. Mice that were exposed to VPA and treated with vehicle exhibited a decrease in social interaction compared with control mice that were treated with vehicle. Rapamycin treatment in VPA-exposed mice improved social deficits. Mice that were exposed to VPA and treated with vehicle exhibited the aberrant expression of genes in the mTOR signaling pathway, and rapamycin treatment recovered changes in the expression of some genes, including *Fyb* and *A330094K24Rik*. Rapamycin treatment suppressed S6 phosphorylation in VPA-exposed mice. Aberrant gene expression was associated with social interaction deficits in VPA-exposed mice. Rapamycin may be an effective treatment for non-syndromic ASD in adolescent and adult patients who present impairments in the mTOR signaling pathway.

**Electronic supplementary material:**

The online version of this article (10.1186/s13041-018-0423-2) contains supplementary material, which is available to authorized users.

## Introduction

Autism spectrum disorder (ASD) is a neurodevelopmental disorder that is characterized by deficits in social interaction and communication, repetitive behaviors, and restricted interests [1]. Autism spectrum disorder has several peripheral symptoms (e.g., aberrant sensitization, clumsiness of movement, and digestive system disease), but social interaction deficits are a core symptom [2]. The prevalence of ASD in school-aged children is > 1–2% [3–5], with a male/female ratio of approximately 4:1 [6]. Various genetic and/or environmental factors have been investigated in studies of ASD using animal models of syndromic ASD and non-syndromic ASD [7]. However, the pathophysiology of social interaction deficits in ASD and effective treatments have not been clarified.

Mammalian target of rapamycin (mTOR) is a serine/threonine kinase that belongs to the family of phosphatidylinositol-3 kinase (PI3K)-related kinases (PIKKs) [8–10]. The mTOR signaling pathway plays a crucial role in protein synthesis and cell growth, proliferation, and metabolism [10]. Overactivation of the mTOR signaling pathway causes diseases that are syndromic with ASD, such as tuberous sclerosis complex (TSC), neuronal fibromatosis 1 (NF1), and tensin homolog deleted on chromosome 10 (PTEN) [11]. Approximately 50% of child and adolescent patients with TSC are diagnosed with ASD [12]. *TSC1*, *TSC2*, *PTEN*, and *NF1* are negative regulators of mTORC1, and mice that possess a mutation of these genes are considered animal models of syndromic ASD [13]. *Tsc1*^*+/−*^ mice exhibit impairments in learning and memory that depend on the hippocampus and a reduction of social interaction compared with wild-type mice [14]. Disturbances of excitatory/inhibitory synaptic balance are thought to be involved in the pathology of ASD [15]. Hippocampal hyperexcitability has been reported in *Tsc1* knockout (KO) cultures, and *Tsc1* conditional KO mice exhibited elevations of S6 phosphorylation in the hippocampus compared with wildtype mice [16]. *PTEN* KO and *Nf1*^*+/−*^ mice also exhibit social interaction deficits [17–19]. Rapamycin improved learning and memory deficits in *Tsc2*^*+/−*^ mice [20]. Rapamycin treatment also recovered social interaction deficits in *Tsc1*^*+/−*^ and *Tsc2*^*+/−*^ mice and rescued the levels of phosphorylated S6K, which is downstream of the mTOR signaling pathway and involved in protein synthesis in *Tsc2*^*+/−*^ mice [21]. Treatment with rapamycin improved social interaction deficits and spine pruning defects in *Tsc2*^*+/−*^ mice [22]. Rapamycin administration in *PTEN* KO mice also attenuated anxiety-like behavior, attenuated sociability deficits, and increased the ratio of Akt phosphorylation [23]. Furthermore, a recent clinical study reported that everolimus, an mTOR inhibitor, ameliorated autistic behavior scores in a patient with TSC [24]. These studies suggest that overactivation of the mTOR signaling pathway is associated with ASD, and mTOR inhibition may be a potential therapeutic strategy for the treatment of syndromic ASD. However, unclear is whether rapamycin is effective for the treatment of non-syndromic ASD.

Valproic acid (VPA) is used as an anti-epileptic drug, mood stabilizer, and treatment for migraine. However, pregnant mothers who are treated with VPA have been reported to deliver children with fetal valproate syndrome and a high incidence of ASD [25]. Valproic acid is used to model non-syndromic ASD in animals. Valproic acid-treated mice and rats exhibit impairments in motor function, aberrant sensitivity, and social interaction deficits [26–28]. Valproic acid activated the PI3K/Akt/mTOR pathway in muscle in a mouse model of Duchenne muscular dystrophy [29]. A reduction of PTEN protein levels and a higher ratio of Akt phosphorylation were found in VPA-exposed rat brains [30]. The blockade of *N*-methyl-D-aspartate (NMDA) receptors, which are upstream of the mTOR signaling pathway, attenuated social interaction deficits in VPA-exposed mice [31]. Nicolini et al. reported that the phosphorylation of mTOR, Akt, and S6 decreased in the lateral temporal neocortex in VPA-exposed rats compared with saline-treated rats [32]. A recent study reported that rapamycin treatment suppressed hippocampal neuron apoptosis in VPA-exposed rats [33]. Furthermore, rapamycin treatment attenuated social interaction deficits and the enhancement of mTOR and S6 phosphorylation in the cerebellum, prefrontal cortex, and hippocampus in VPA-exposed rats on postnatal day 33–35 (adolescence) [34]. These previous studies suggest that an aberrant mTOR signaling pathway causes ASD-like behaviors in VPA-exposed animals. However, the effects of rapamycin on social interaction deficits have not been investigated in adult mice that were exposed to VPA in utero. Moreover, gene expression analysis in whole brains has not been performed in VPA-exposed or rapamycin-treated mice. Therefore, the present study investigated the effect of rapamycin treatment on social interaction deficits in adolescent and adult mice that were exposed to VPA in utero. We also comprehensively analyzed the gene expression including mTOR signaling pathway and S6 phosphorylation in mouse whole brains.

## Methods

### Animals and VPA administration

Pregnant female C57BL/6 J mice (CLEA, Tokyo, Japan) received a single subcutaneous injection of 600 mg/kg sodium valproate (Sigma-Aldrich, St. Louis, MO, USA) on day 12.5 after conception. Valproic acid was dissolved in saline, and control mice received saline. All of the mice were returned to their home cages immediately after the injection. We used 10 dams. Eight to 10 pups were obtained from VPA- and saline-treated pregnant female mice. The pups were culled to eight animals per litter on P4. The number of mice per litter was normal compared with control mice. In this experiment, we did not observe postnatal mortality. The day of birth was recorded as day 0, and all of the pups were labeled for individual identification. The pups were weaned, sexed, and caged in groups of 3–5 mice of the same sex on postnatal day 26 (P26). All of the behavioral tests were conducted from 9:00 AM to 6:00 PM. The mice were housed on a 12 h/12 h light/dark cycle (lights on 8:00 AM to 8:00 PM), and temperature was maintained at 22 °C. All of the mice had ad libitum access to food and water. In the present study, we analyzed male mice only because the prevalence of ASD is higher in males than in females. All of the animal experiments were performed in accordance with the Guidelines for the Care of Laboratory Animals of the Tokyo Metropolitan Institute of Medical Science, and the housing conditions were approved by the Institutional Animal Care and Use Committee.

### Behavioral tests

**Fig. 1:**
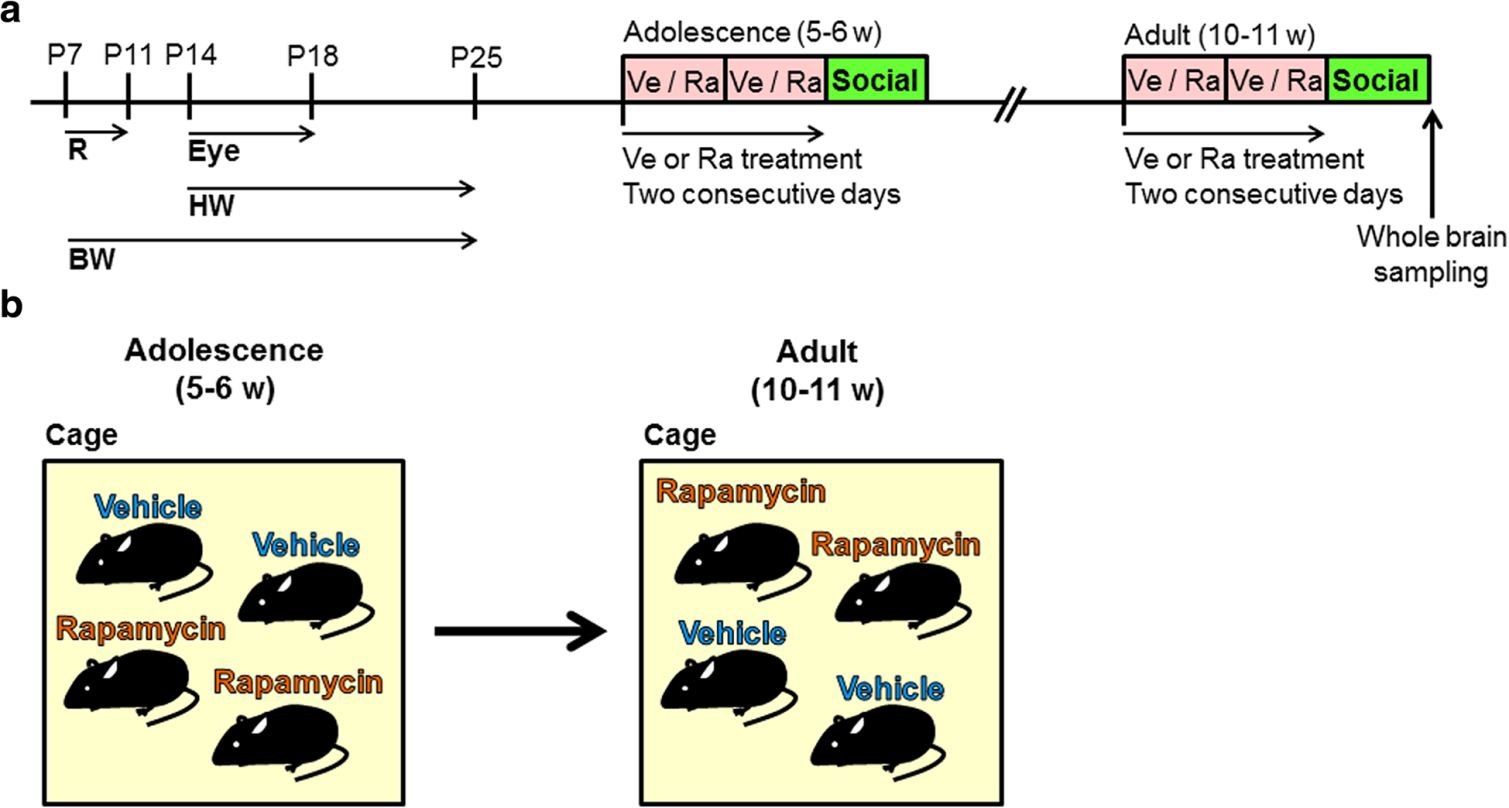
Schedule of experimental design and rapamycin treatment. (**a**) Body maturation and motor function were assessed from P7 to P25. The social interaction test was conducted in both adolescence (5–6 weeks of age) and adulthood (10–11 weeks of age). The mice were scarified to sample whole brains. R, righting reflex test; Eye, eye-opening test; HW, hanging wire test; BW, body weight; Ra, rapamycin; Ve, vehicle; Social, social interaction test. (**b**) The mice were reared with their littermates. A mouse that was injected with vehicle in adolescence was injected with rapamycin in adulthood. Conversely, a mouse that was injected with rapamycin in adolescence was injected with vehicle in adulthood

Figure 1a shows a schedule of the experiments. The behavioral tests were conducted in a sound-proof room, and the mice were given a 60 min habituation period after transportation to the behavioral room before the start of each test. Body maturation was assessed by measuring body weight and eye-opening. Motor function was assessed by the righting reflex and hanging wire tests. The social interaction test was conducted in both adolescence (5–6 weeks of age) and adulthood (10–11 weeks of age).

#### Body weight and eye-opening test

Body weight was recorded on P7, P11, P14, P18, P21, and P25. Body weight was also recorded when each mouse underwent the social interaction test in both adolescence and adulthood. Eye opening was observed once daily from P14 to P18. The eye-opening score was the following: 0 = both eyes closed, 1 = one eye open, and 2 = both eyes open.

#### Righting reflex test

Each mouse was placed on its back and gently held by its four limbs. The latency to right, such that all four paws were touching the surface, was recorded. The cutoff time was set at 20 s. Each mouse underwent the righting reflex test on P7, P9, and P11.

#### Hanging wire test

The hanging wire test (O’Hara & Co., Tokyo, Japan) was conducted from P14 to P25. The mice were placed on a grid wire surface (150 mm × 150 mm, divided into 10 mm grid squares). The plane was inverted, and the latency to fall was recorded, with a maximum of 600 s.

#### Social interaction test

We conducted the social interaction test as previously described [21]. Each mouse was left alone in its home cage for 15 min for habituation. The home cage was placed in a sound-attenuating chamber. An unfamiliar C57BL/6 J mouse of the same sex and age was then introduced. The behavior of the test mouse was video-recorded for 10 min and blindly scored for active social interaction, consisting of sniffing, allo-grooming, mounting, and following. A mouse, which went out of its home cage during the 15 min habituation period, was excluded from analysis. Each mouse underwent the social interaction test during both adolescence and adulthood. One of the 10 rapamycin-treated vehicle mice and one of the 11 rapamycin-treated VPA mice went out of their home cages for a habituation period during adolescence. The number of mice per group was the following: adolescent (*n* = 11 vehicle-treated control mice, *n* = 13 vehicle-treated VPA-exposed mice, *n* = 9 rapamycin-treated control mice, *n* = 10 rapamycin-treated VPA-exposed mice) and adult (*n* = 10 vehicle-treated control mice, *n* = 11 vehicle-treated VPA-exposed mice, *n* = 11 rapamycin-treated control mice, *n* = 13 rapamycin-treated VPA-exposed mice).

### Rapamycin treatment

Rapamycin (LC Laboratories, Woburn, MA, USA) was dissolved in 10% dimethyl sulfoxide diluted with saline. The mice received a 10 ml/kg rapamycin solution or an equal volume of vehicle intraperitoneally once daily for 2 consecutive days. The dose of rapamycin was 10 mg/kg. Brain levels of rapamycin remained sufficiently high to inhibit mTOR throughout the 48 h period after rapamycin administration (6 mg/kg, i.p.) in mice [35]. Sato et al. reported that 5 or 10 mg/kg rapamycin treatment effectively attenuated social interaction deficits in *Tsc1*^*+/−*^ and *Tsc2*^*+/−*^ mice [21]. Thus, we tested the dose of 10 mg/kg rapamycin. The social interaction test was performed 24 h after the second administration. Each mouse was randomly assigned to vehicle or rapamycin administration in adolescence (Fig. 1b). A mouse that received vehicle in adolescence received rapamycin in adulthood. Conversely, a mouse that received rapamycin in adolescence received vehicle in adulthood.

### Brain collection and RNA extraction

Whole brains were collected after the end of the social interaction test in adulthood. Because the precise brain regions that are associated with ASD have not yet been fully clarified, we examined whole brains in the present study. Brains were frozen in liquid nitrogen and stored at − 80 °C until further processing. Total RNA that was extracted from whole brains was homogenized in Ambion TRIzol Reagent (Thermo Fisher Scientific, Waltham, MA, USA) using a homogenizer. RNA was isolated using chloroform and precipitated using isopropyl alcohol. The quality of RNA was assessed with Nanodrop 1000 (Thermo Fisher Scientific). All of the RNA samples had an A_260/280_ ratio between 2.0 and 2.1 and an A_230/260_ ratio between 2.2 and 2.3.

### Microarray analysis

cRNA targets were synthesized and hybridized using the Whole Mouse Genome Microarray according to the manufacturer’s instructions (Agilent Technologies, Santa Clara, CA, USA). The array slides were scanned using a SureScan Microarray Scanner (Agilent Technologies). Before analyzing gene expression, microarray data were normalized and sorted using GeneSpring 14.5 software (Agilent Technologies). Each sample was normalized by a 75% percentile shift. Compromised probes were removed, and remaining probes with expression values < 20% were excluded. The probes were then filtered based on expression levels for quality control. The Benjamini and Hochberg false-discovery rate (FDR) was determined for the remaining probes and those with *p* < 0.05. Each group comparison was performed using *t*-tests (*p* < 0.05). Each group consisted of five mice (vehicle-treated control mice, rapamycin-treated control mice, vehicle-treated VPA-exposed mice, and rapamycin-treated VPA-exposed mice).

### Mining of public databases

The genomic data repositories in BaseSpace (illumina, https://www.nextbio.com/b/authentication/login.nb) were used to analyze all differentially expressed genes with statistical significance from vehicle-treated VPA-exposed mice vs. vehicle-treated control mice, rapamycin-treated VPA-exposed mice vs. vehicle-treated VPA-exposed mice, and rapamycin-treated control mice vs. vehicle-treated control mice. The data were compared with curated datasets that are available in BaseSpace to identify published studies of Diseases, Pharmaco, and Knockout mice using the BaseSpace Diseases atlas application, BaseSpace Pharmaco atlas application, and BaseSpace Knockout atlas application, respectively. Rank-based enrichment statistics were employed to calculate BaseSpace scores for each disease, compound, and gene for knockout mice. MetaCore (Thomson Reuters, https://portal.genego.com) was used to build the network for two negative correlation genes in vehicle-treated VPA-exposed mice and vehicle-treated control mice vs. rapamycin-treated VPA-exposed mice and vehicle-treated VPA-exposed mice.

### Antibodies and Western blot

Rabbit anti-S6 antibodies and antiphospho-S6 (S235/236) antibodies (1:500) were purchased from Cell Signaling Technology (Danvers, MA, USA). We conducted protein extraction and Western blot as previously described [21]. We prepared protein samples from the whole brain because the precise brain regions that are associated with ASD have not yet been fully clarified. Total protein from the frozen mouse brain was extracted for Western blot. Whole brains were homogenized using a tissue homogenizer in 1× sodium dodecyl sulfate (SDS) gel-loading buffer (50 mM Tris–HCl [pH 6.8], 2% SDS, and 10% glycerol). The supernatant was obtained by centrifugation at 17,000×*g*. The protein concentration was determined using the Bio-Rad DC Protein Assay Kit (Bio-Rad Laboratories, Hercules, CA, USA). Equal amounts of extracted protein were added to 5% mercaptoethanol and boiled. Proteins were resolved by SDS-polyacrylamide gel electrophoresis (PAGE), transferred to a polyvinylidene fluoride membrane (Immobilon-P, Merck Millipore, Billerica, MA, USA), and blocked in 1% skim milk/Tris-buffered saline that contained 0.05% Tween 20 at room temperature for 2 h. The membranes were incubated with the primary antibodies at room temperature for 1 h. Protein bands were detected using the EnVisiont Kit (Dako, Glostrup, Denmark) and ECL Western Blotting Detection System (GE Healthcare, Buckinghamshire, UK) and quantitatively analyzed using ImageJ 1.45 software.

### Statistical analysis

The results of the behavioral tests were analyzed using Statistical Package for the Social Sciences 14.0 software (SPSS, Tokyo, Japan). The data were analyzed using Student’s *t*-test, the Mann-Whitney *U* test, and two-way analysis of variance (ANOVA). All of the data are presented as mean ± standard error of the mean (SEM). Values of *p* < 0.05 were considered statistically significant.

## Results

### Body maturation and motor function

**Fig. 2:**
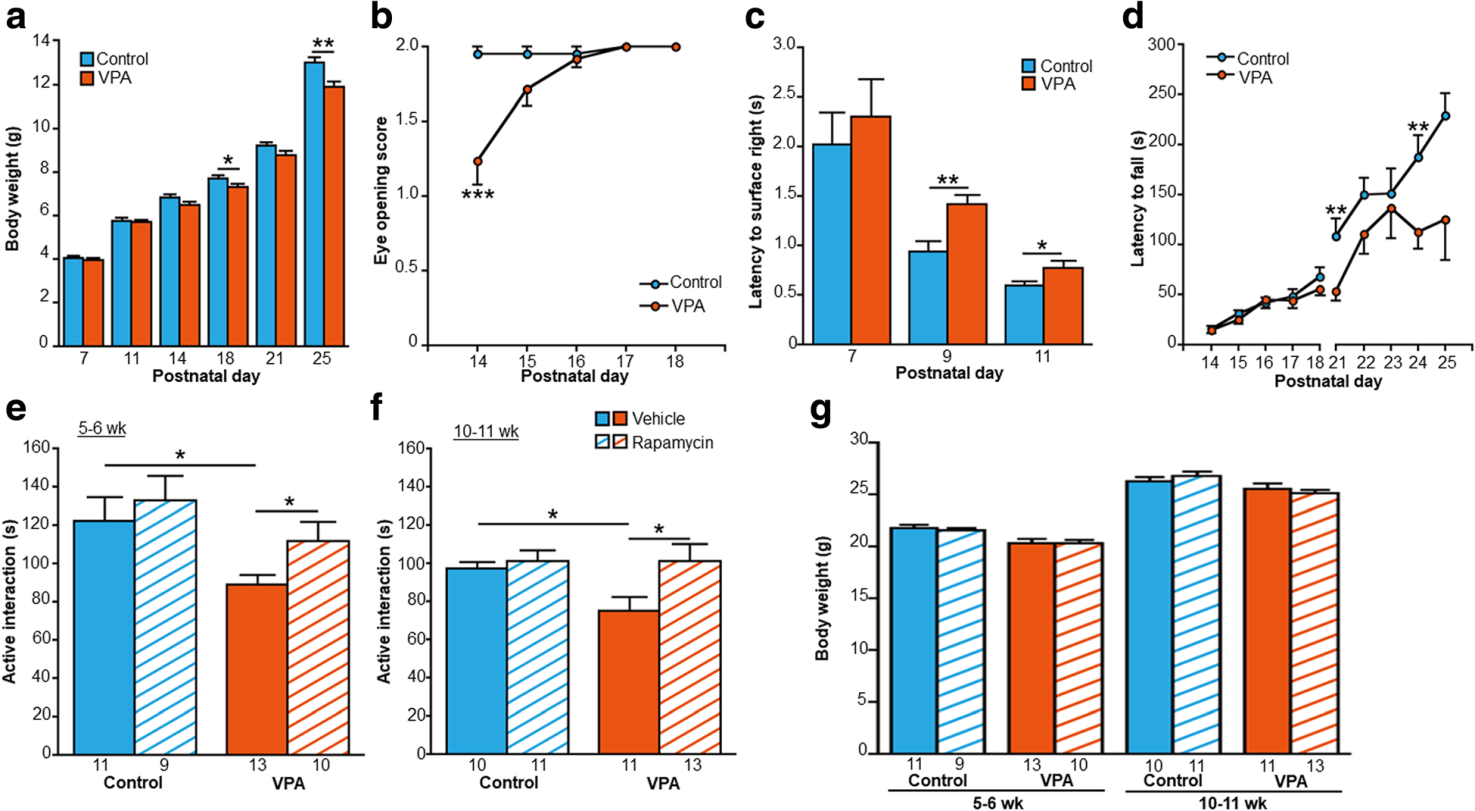
Body maturation, motor function, and effects of rapamycin treatment on social interaction and body weight. (**a**) Valproic acid-exposed mice exhibited lower body weight on P18 and P25. (**b**) Valproic acid-exposed mice had lower eye-opening scores on P14. (**c**) Valproic acid-exposed mice exhibited a longer latency to right on P9 and P11 in righting reflex test. (**d**) Valproic acid-exposed mice exhibited a shorter latency to fall from P21 to P25 in the hanging wire test. (**e**) Social interaction test (5–6 weeks of age). Vehicle-treated VPA-exposed mice exhibited a decrease in active social interaction time compared with vehicle-treated control mice. Rapamycin-treated VPA-exposed mice exhibited an increase in active social interaction time compared with vehicle-treated VPA-exposed mice. (**f**) Social interaction test (10–11 weeks of age). Vehicle-treated VPA-exposed mice exhibited a decrease in active social interaction time compared with vehicle-treated control mice. Rapamycin-treated VPA-exposed mice exhibited an increase in active social interaction time compared with vehicle-treated VPA-exposed mice. (**g**) No significant difference in body weight was found between vehicle treatment and rapamycin treatment. Administration of rapamycin for 2 consecutive days did not affect body weight. Significant differences in body weight were found between control and VPA-exposed mice in both adolescence and adulthood. Error bars indicate SEM. *p < 0.05, **p < 0.01, ***p < 0.001

The two-way ANOVA showed significant main effects of VPA treatment (*F*_1,43_ = 5.522, *p* = 0.023) and postnatal day (*F*_2.026,43_ = 1639.1, *p* = 0.000) and a significant VPA treatment × postnatal day interaction (*F*_2.026,43_ = 1639.1, *p* = 0.000; *n* = 21 control mice, *n* = 24 VPA-exposed mice; Fig. 2a). The Bonferroni post hoc test showed a significant reduction of body weight in VPA-exposed mice on P18 and P25 (P18: *p* = 0.032; P25: *p* = 0.002). Eye opening scores in VPA-exposed mice were lower than in control mice on P14 (*U* = 125, *p* = 0.000; *n* = 21 control mice, *n* = 24 VPA-exposed mice), with no significant difference between control and VPA-exposed mice on P15 (*p* = 0.114), P16 (*p* = 0.924), P17 (*p* = 1.000), and P18 (*p* = 1.000; Fig. 2b). In the righting reflex test (Fig. 2c), the two-way ANOVA showed no main effect of VPA treatment (*F*_1,42_ = 2.471, *p* = 0.123) and no VPA treatment × postnatal day interaction (*F*_2.026,42_ = 0.294, *p* = 0.620; *n* = 21 control mice, *n* = 24 VPA-exposed mice). Both groups (i.e., exposed to VPA or saline) exhibited a progressive decline in the latency to right on P7, P9, and P11 (*F*_2.026,42_ = 0.294, *p* = 0.620). The two-way ANOVA showed no main effect of VPA treatment (*F*_1,24_ = 0.371, *p* = 0.548) and no VPA treatment × postnatal day interaction (*F*_3.054,24_ = 0.355, *p* = 0.790). The Bonferroni post hoc test showed a significantly longer latency in VPA-exposed mice on P9 and P11 (P9: *p* = 0.001; P11: *p* = 0.046). Both groups (i.e., exposed to VPA or saline) exhibited a progressive increase in the latency to fall on P14–18 (*F*_3.054,24_ = 16.621, *p* = 0.000; Fig. 2d). The two-way ANOVA revealed a significant main effect of VPA treatment on the latency to fall on P21–25 (*F*_1,22_ = 12.250, *p* = 0.002), with no main effect of postnatal day (*F*_2.835,22_ = 2.703, *p* = 0.056) and no VPA treatment × postnatal day interaction (*F*_2.835,22_ = 0.626, *p* = 0.592). The Bonferroni post hoc test showed a significantly shorter latency to fall in VPA-exposed mice on P21 and P24 (P21: *p* = 0.001; P24: *p* = 0.001).

We assessed the effects of rapamycin treatment on social interaction in control and VPA-exposed mice during both adolescence and adulthood. In adolescence (Fig. 2e), vehicle-treated VPA-exposed mice exhibited a decrease in active social interaction time compared with vehicle-treated control mice (*t*_22_ = 2.669, *p* = 0.014; *n* = 11 vehicle-treated control mice, *n* = 13 vehicle-treated VPA-exposed mice). Active social interaction time in rapamycin-treated VPA-exposed mice significantly increased compared with vehicle-treated VPA-exposed mice (*t*_21_ = − 2.134, *p* = 0.045; *n* = 13 vehicle-treated VPA-exposed mice, *n* = 10 rapamycin-treated VPA-exposed mice). No significant difference in active social interaction time was found between vehicle-treated control mice and rapamycin-treated control mice (*t*_18_ = − 0.599, *p* = 0.557; *n* = 11 vehicle-treated control mice, *n* = 9 rapamycin-treated control mice). In adulthood (Fig. 2f), vehicle-treated VPA-exposed mice exhibited a decrease in active social interaction time compared with vehicle-treated control mice (*t*_19_ = 2.586, *p* = 0.018; *n* = 10 vehicle-treated control mice, *n* = 11 vehicle-treated VPA-exposed mice). Rapamycin-treated VPA-exposed mice exhibited an increase in active social interaction time compared with vehicle-treated VPA-exposed mice (*t*_22_ = − 2.284, *p* = 0.032; *n* = 11 vehicle-treated VPA-exposed mice, *n* = 13 rapamycin-treated VPA-exposed mice). No significant difference in active social interaction time was found between vehicle-treated control mice and rapamycin-treated control mice (*t*_21_ = − 0.415, *p* = 0.682; *n* = 10 vehicle-treated control mice, *n* = 11 rapamycin-treated control mice). Body weight was recorded immediately before the social interaction test (Fig. 2g). A significant difference in body weight was found between control and VPA-exposed mice in adolescence (5–6 weeks of age; vehicle-treated control mice vs. vehicle-treated VPA-exposed mice, *t*_22_ = 3.226, *p* = 0.004; rapamycin-treated control mice vs. rapamycin-treated VPA-exposed mice, *t*_19_ = − 3.255, *p* = 0.004), with no significant difference between vehicle-treated control mice vs. rapamycin-treated control mice (*t*_19_ = 0.610, *p* = 0.549) or between vehicle-treated VPA-exposed mice vs. rapamycin-treated VPA-exposed mice (*t*_22_ = 0.617, *p* = 0.869). In adulthood (10–11 weeks of age), rapamycin-treated VPA-exposed mice exhibited lower body weight than rapamycin-treated control mice (*t*_20.846_ = − 2.941, *p* = 0.008). No significant difference in body weight was found between vehicle-treated control mice and vehicle-treated VPA-exposed mice (*t*_19_ = 1.005, *p* = 0.327). No significant difference in body weight was found between vehicle-treated control mice vs. rapamycin-treated control mice (*t*_19_ = − 0.899, *p* = 0.380) or between vehicle-treated VPA-exposed mice vs. rapamycin-treated VPA-exposed mice (*t*_22_ = 0.753, *p* = 0.459).

### Effects of VPA exposure in utero on gene expression

Vehicle-treated VPA-exposed mice (*n* = 5) exhibited the differential expression of 5644 genes (2761 upregulated genes, 2883 downregulated genes) compared with vehicle-treated control mice (*n* = 5; Table 1; for information for each gene, see Additional file 1: Table S1a). We detected associations between the up- and downregulated genes in VPA-exposed mice and curated studies of Diseases, Compounds, and Knockout mice in the BaseSpace database. As shown in Additional file 2: Table S2a, we found that 148 diseases were positively correlated with upregulated genes using the BaseSpace Diseases atlas application. Vitamin D deficiency, which is a Nutritional and Metabolic Disease, had the highest score (96.94107042). Within the top five in the score list, we found Developmental Disorder and Developmental Delay (score = 82.71872854). We found that 136 diseases were positively correlated with downregulated genes using the BaseSpace Diseases atlas application. Injury of the eye region, which is an Eye Disorder, had the highest score (92.03862769). The BaseSpace Pharmaco atlas application showed that 387 compounds were positively correlated with upregulated genes (Additional file 2: Table S2b). Glycidol had the highest score (100). The BaseSpace Pharmaco atlas application showed that 446 compounds were positively correlated with downregulated genes. Ozagrel had the highest score (84.66459954). The BaseSpace Knockout atlas application showed that 26 KO mice were positively correlated with upregulated genes (Additional file 2: Table S2c). PRKG1 had the highest score (93.20344653). The BaseSpace Knockout atlas application showed that 25 KO mice were positively correlated with downregulated genes. PRKG1 had the highest score (79.58880595).

**Table 1:** Number of genes with altered expression in each group

Group	Total altered genes	Upregulated genes	Downregulated genes
VPA + vehicle/Control + vehicle	5644	2761	2883
VPA + rapamycin/VPA + vehicle	23	7	16
Control + rapamycin/Control + vehicle	–	–	–

### Expression of genes associated with mTOR signaling pathway

We found 5644 significantly altered genes (2761 upregulated genes, 2883 downregulated genes) between vehicle-treated control mice (control + vehicle) and vehicle-treated VPA-exposed mice (VPA + vehicle; Table 1). Twenty-three genes (seven upregulated genes, 16 downregulated genes; for information for each gene, see Additional file 1: Table S1b) were differentially expressed in rapamycin-treated VPA-exposed mice (VPA + rapamycin, *n* = 5) compared with vehicle-treated VPA-exposed mice (VPA + vehicle, *n* = 5). No significantly altered genes were found between rapamycin-treated control mice (control + rapamycin, *n* = 5) and vehicle-treated control mice (control + vehicle, *n* = 5). Altered genes that are associated with the mTOR signaling pathway between vehicle-treated control mice and vehicle-treated VPA-exposed mice were extracted from Pathway Enrichment of BaseSpace (Table 2). *mTOR*, *Mapk1*, *Rps6ka3*, *Akt2*, and *Pick3cg* had fold changes > 1.2 or < − 1.2. These genes in the mTOR pathway (Table 2) were unaltered between vehicle-treated VPA-exposed mice and rapamycin-treated VPA-exposed mice.

**Table 2:** Expression change in mTOR signaling pathway-associated genes in vehicle-treated VPA-exposed mice and vehicle-treated control mice

Gene	EntrezGene ID	Imported ID	Fold change
*Mtor*	56,717	A_52_P67643	−1.5175
*Mapk1*	26,413	A_55_P1973339	−1.2254
*Rheb*	19,744	A_55_P2129034	−1.1928
*Rps6kb1*	72,508	A_55_P2019083	−1.173
*Cab39*	12,283	A_51_P230934	−1.163
*Pld2*	18,806	A_55_P2049572	−1.1607
*Eif4a1*	13,681	A_55_P2046398	−1.158
*Yy1*	22,632	A_55_P2107745	−1.1493
*Cab39l*	69,008	A_55_P2001948	−1.1442
*Rheb*	19,744	A_55_P2170139	−1.1396
*Atg13*	51,897	A_55_P2028394	−1.135
*Tsc1*	64,930	A_55_P2157695	−1.132
*Pik3cd*	18,707	A_52_P99848	−1.1195
*Ppp2ca*	19,052	A_55_P2288117	−1.1053
*Raf1*	110,157	A_55_P1973643	−1.09
*Ulk1*	22,241	A_55_P1977314	−1.084
*Pik3r3*	18,710	A_55_P2000158	−1.0813
*Rhoa*	11,848	A_55_P2008081	−1.0709
*Rac1*	19,353	A_55_P2076489	−1.0697
*Ywhaq*	22,630	A_55_P2142296	−1.0651
*Fbxw11*	103,583	A_51_P141860	−1.0642
*Mapkap1*	227,743	A_55_P2046363	1.0597
*Map2k1*	26,395	A_51_P241074	1.0611
*Eif4g2*	13,690	A_51_P306066	1.0807
*Eif4g3*	230,861	A_55_P2034372	1.0837
*Rps6*	20,104	A_55_P1955239	1.0851
*Rac1*	19,353	A_51_P513254	1.0907
*Ywhaq*	22,630	A_65_P16952	1.0955
*Eif4g1*	208,643	A_55_P2046378	1.1071
*Rragc*	54,170	A_51_P346826	1.1071
*Stk11*	20,869	A_55_P2166399	1.1071
*Eif4b*	75,705	A_51_P151271	1.1223
*Clip1*	56,430	A_55_P2006519	1.1358
*Vegfa*	22,339	A_52_P638895	1.1535
*Vegfb*	22,340	A_52_P436628	1.155
*Eif4ebp1*	13,685	A_51_P330428	1.1939
*Vegfa*	22,339	A_52_P249424	1.1976
*Rps6ka3*	110,651	A_55_P2404434	1.2021
*Akt2*	11,652	A_55_P2066523	1.2491
*Pik3cg*	30,955	A_51_P507832	1.3404

### Characteristics of gene expression induced by rapamycin treatment

**Fig. 3:**
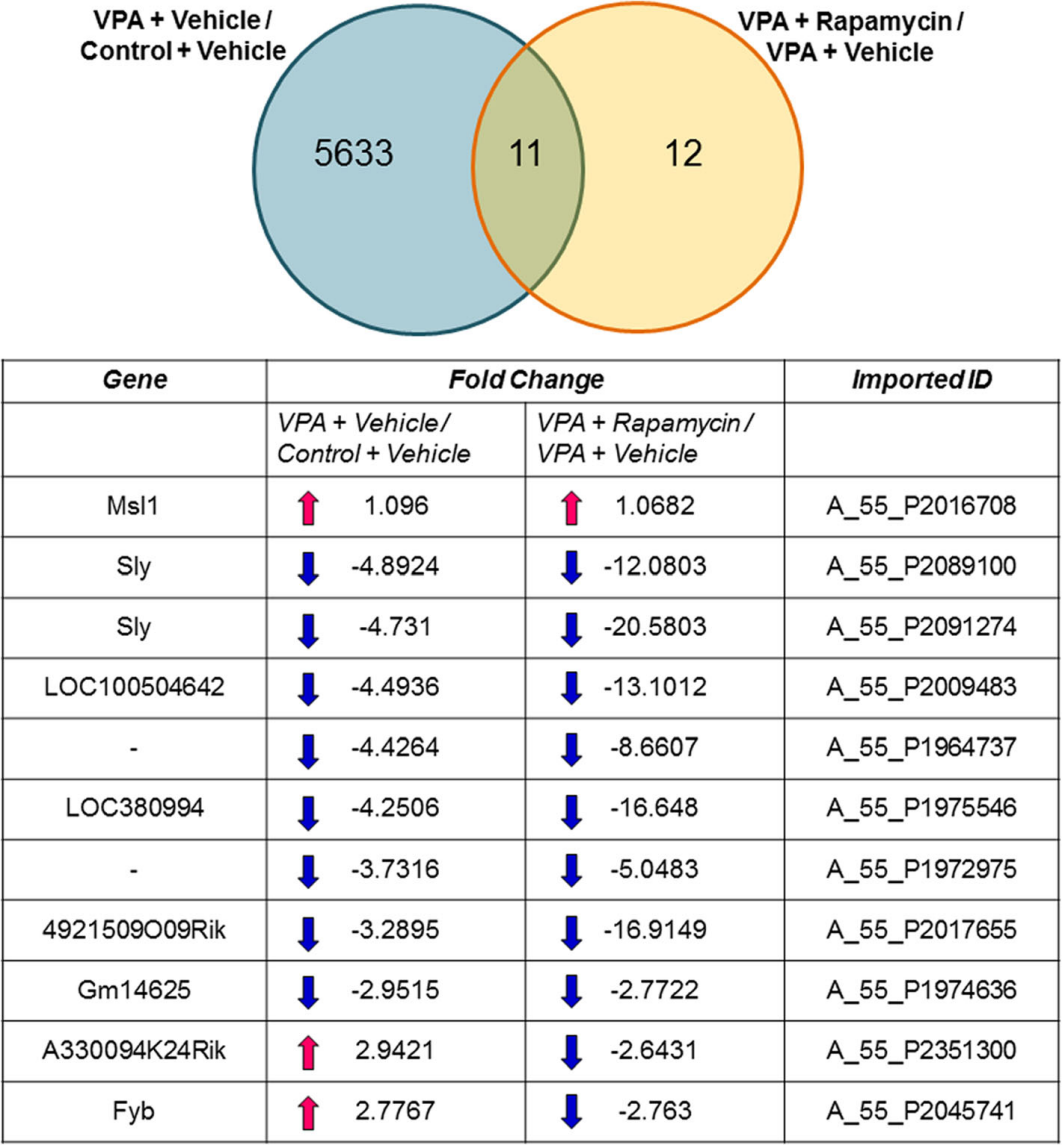
Effects of rapamycin treatment on gene expression. The expression of 5644 genes significantly changed between vehicle-treated VPA-exposed mice and vehicle-treated control mice (VPA + vehicle / control + vehicle). The expression of 23 genes significantly changed between vehicle-treated VPA-exposed mice and rapamycin-treated VPA-exposed mice (VPA + rapamycin / VPA + vehicle). The expression of 11 genes changed in both groups. The expression of *A330094K24Rik* and *Fyb* oppositely changed between the two groups

**Fig. 4:**
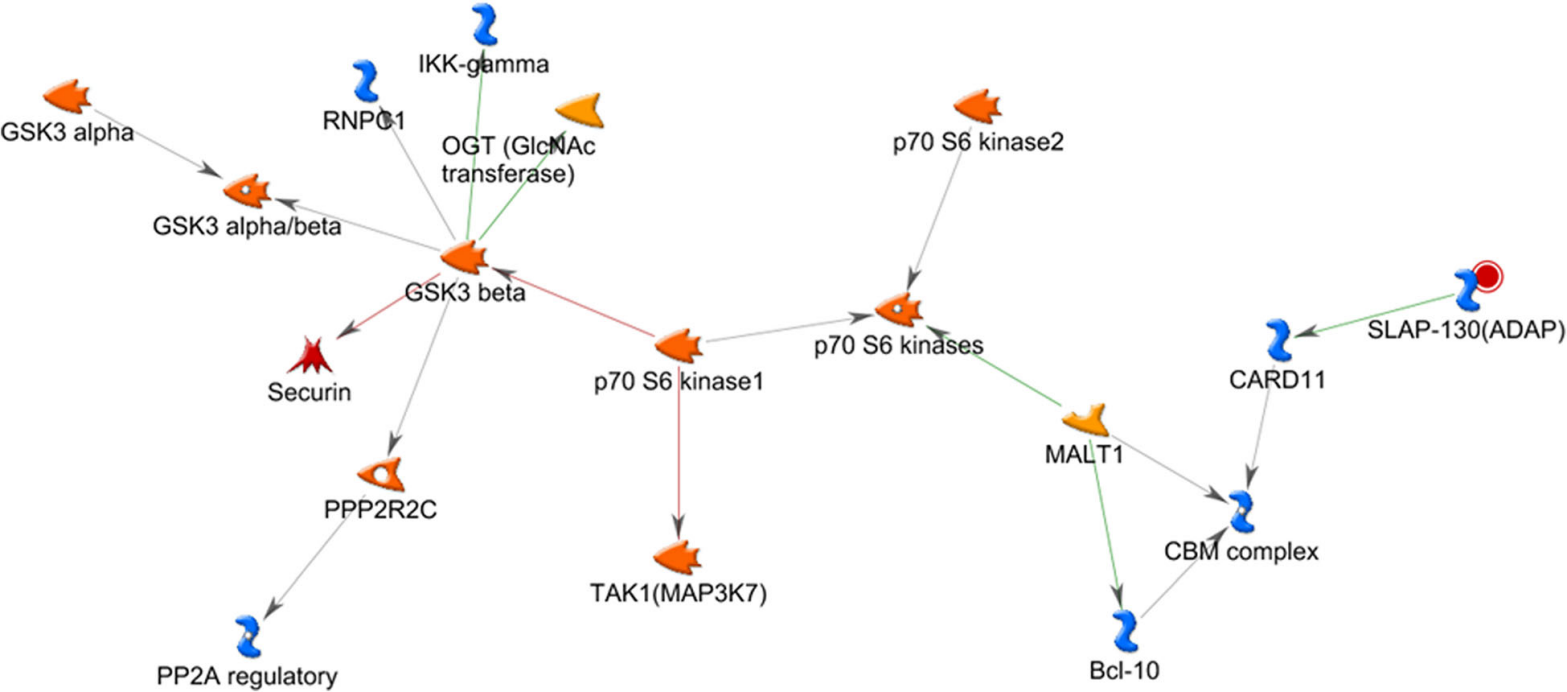
MetaCore network built from *Fyb* (SLAP-130 or ADAP). *Fyb* is also referred to as SLAP-130 or ADAP. The red circle shows *Fyb* (SLAP-130 or ADAP). The networks that are associated with *Fyb* were built using “build networks” in MetaCore, and interactions were visualized. In these networks, *Fyb* is indirectly related to S6K (p70 S6 kinase). Bcl-10, B-cell CLL/lymphoma 10; CARD11, caspase recruitment domain family member 11; CBM complex, CARD11-Bcl-10-MALT1 signalosome complex; GSK3 alpha, glycogen synthase kinase 3α; GSK3 alpha/beta, glycogen synthase kinase 3α/β; GSK3 beta, glycogen synthase kinase 3β; IKK-gamma, inhibitor of nuclear factor κB kinase subunit γ; MALT1, MALT1 paracaspase; OGT (GlcNAc transferase), *O*-linked *N*-acetylglucosamine transferase; p70 S6 kinase, 70 KDa ribosomal protein S6 kinase; p70 S6 kinase 1, 70 KDa ribosomal protein S6 kinase 1; p70 S6 kinase 2, 70 KDa ribosomal protein S6 kinase 2; PP2A regulatory, protein phosphatase 2A; PPP2R2C, protein phosphatase 2 regulatory subunit Bγ; RNPC1, RNA binding motif protein 38 (RNPC1 is previous HGNC symbol for RBM38 gene); Securin, Securin is also referred to as pituitary tumor-transforming 1 (PTTG1); TAK1 (MAP3K7), mitogen-activated protein kinase kinase kinase 7 (TAK1 is previous HGNC symbol for MAP3K7)

Figure 3 presents a Venn diagram of significantly altered gene expression in vehicle-treated VPA-exposed mice (VPA + vehicle) vs. vehicle-treated control mice (control + vehicle; 5644 genes) and rapamycin-treated VPA-exposed mice (VPA + rapamycin) vs. vehicle-treated VPA-exposed mice (VPA + vehicle; 23 genes). We found 11 common genes (*Msl1*, *Sly*, *LOC100504642*, *LOC380994*, *4921509O09Rik*, *Gm14625*, *A330094K24Rik*, *Fyb*, two genes that have no gene symbols, and *Sly* that has two probes; Fig. 3). *Msl1* expression increased in both vehicle-treated VPA-exposed mice vs. vehicle-treated control mice (VPA + vehicle/control + vehicle) and rapamycin-treated VPA-exposed mice vs. vehicle-treated VPA-exposed mice (VPA + rapamycin/VPA + vehicle). *Sly*, *LOC100504642*, *LOC380994*, *4921509O09Rik*, *Gm14625*, and two genes without gene symbols decreased in both vehicle-treated VPA-exposed mice vs. vehicle-treated control mice (VPA + vehicle / control + vehicle) and rapamycin-treated VPA-exposed mice vs. vehicle-treated VPA-exposed mice (VPA + rapamycin/VPA + vehicle). *A330094K24Rik* and *Fyb* increased in vehicle-treated VPA-exposed mice vs. vehicle-treated control mice (VPA + vehicle / control + vehicle) but decreased in rapamycin-treated VPA-exposed mice vs. vehicle-treated VPA-exposed mice (VPA + rapamycin / VPA + vehicle). Furthermore, we investigated networks of *A330094K24Rik* and *Fyb* using “build networks” in MetaCore. We were unable to detect networks for *A330094K24Rik* but found that *Fyb* was in networks that are associated with *p70S6*, which is downstream of the mTOR signaling pathway (Fig. 4; *Fyb* is also referred to as SLAP-130 or ADAP).

### Expression and phosphorylation levels of S6

**Fig. 5:**
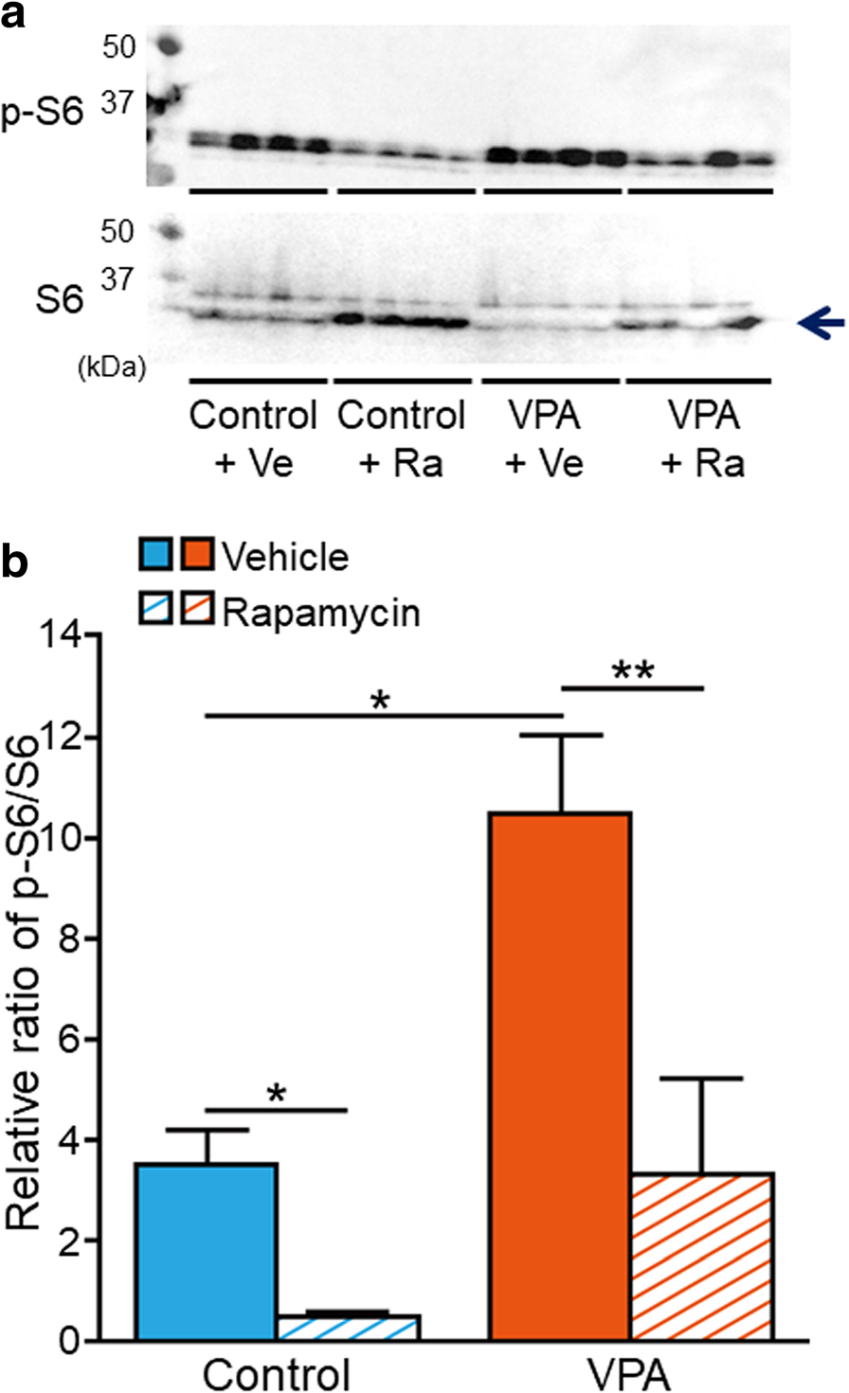
Western blot of S6 protein phosphorylation. (**a**) Immunoblots of phospho- and total S6. Control + Ve; vehicle-treated control mice; Control + Ra, rapamycin-treated control mice; VPA + Ve, vehicle-treated VPA-exposed mice; VPA + Ra, rapamycin-treated VPA-exposed mice. (**b**) Densitometric analysis of S6 proteins. Error bars indicate SEM. *p < 0.05, **p < 0.01

A previous study reported that rapamycin treatment in *Tsc1*^*+/−*^ and *Tsc2*^*+/−*^ mice improved social interaction deficits and decreased the protein level of activated phospho-S6K [21]. The phosphorylation of S6K phosphorylates S6, and this process is associated with protein synthesis [8]. A recent study showed that lymphoblastoid cell lines from patients with idiopathic autism increased S6 phosphorylation compared with controls [36]. Therefore, we analyzed the phosphorylation of S6 protein in whole mouse brains in each group (Fig. 5a). Vehicle-treated VPA-exposed mice exhibited an elevation of phospho-S6 levels compared with vehicle-treated control mice (*t*_6_ = − 4.113, *p* = 0.006; *n* = 4 vehicle-treated control mice, *n* = 4 vehicle-treated VPA-exposed mice; Fig. 5b). Rapamycin-treated VPA-exposed mice exhibited significant suppression of S6 phosphorylation compared with vehicle-treated VPA-exposed mice (*t*_6_ = 2.910, *p* = 0.027; *n* = 4 vehicle-treated VPA-exposed mice, *n* = 4 rapamycin-treated VPA-exposed mice). Rapamycin-treated control mice exhibited significant suppression of S6 phosphorylation compared with vehicle-treated control mice (*t*_6_ = 4.331, *p* = 0.005; *n* = 4 vehicle-treated control mice, *n* = 4 rapamycin-treated control mice).

## Discussion

In the present study, we found that VPA-exposed mice exhibited a delay in body maturation and poor motor performance compared with control mice. We confirmed that rapamycin treatment improved social interaction deficits in VPA-exposed mice, although these improvements in social interaction deficits may be secondary to improvements in other behaviors. We also found that rapamycin treatment for 2 consecutive days improved social interaction deficits without altering body weight in both adolescence and adulthood. Rapamycin treatment reduced the expression of two genes (*Fyb* and *A330094K24Rik*) and S6 protein phosphorylation in vehicle-treated VPA-exposed mice. Furthermore, we found associations between aberrant gene expression in VPA-exposed mice and curated Diseases, Pharmaco, and Knockout mice.

Exposure to VPA in utero in mice and rats has been used to model ASD [37]. Previous studies reported that VPA exposure in utero or in adolescence delayed body maturation and resulted in poor motor performance in both mice and rats [27, 30]. In the present study, we also observed a delay in body maturation and worse motor performance in VPA-exposed mice. These results are consistent with previous studies that reported delays in body maturation and poor motor performance in VPA-exposed mice. An increase or decrease in the expression/phosphorylation of proteins in the mTOR signaling pathway and deficits in social interaction have been reported in VPA-exposed mice and rats. The phosphorylation of Akt, mTOR, 4EBP1, and S6 in the lateral temporal neocortices decreased in VPA-exposed rats [32]. Valproic acid-exposed mice exhibited a decrease in PTEN protein levels and an increase in Akt phosphorylation in the prefrontal cortex and hippocampus on embryonic day 18 and P13 [30]. A recent study reported that VPA-exposed mice exhibited social interaction deficits in the three-chambered social interaction test, a decrease in PTEN protein level, an increase in Akt phosphorylation, and a decrease in the number of Nissl-positive cells in the CA1 area of the hippocampus [38]. These previous results imply that prenatal exposure to VPA causes the aberrant expression/phosphorylation of proteins in the mTOR signaling pathway. In the present study, we also found that vehicle-treated VPA-exposed mice exhibited deficits in social interaction and an increase in S6 protein phosphorylation compared with vehicle-treated control mice. These present results are consistent with previous studies. Rapamycin treatment inhibited apoptosis in the hippocampus in VPA-exposed rats [33]. Qin et al. reported that rapamycin treatment attenuated social interaction deficits and enhanced mTOR and S6 phosphorylation in VPA-exposed rats on P33–35 [34]. These authors also found that treatment with sulindac (an inhibitor of the Wnt signaling pathway) improved social deficits and attenuated the enhancement of mTOR phosphorylation in VPA-exposed rats. Qin et al. suggested that VPA activates both the Wnt and mTOR signaling pathways to induce autism-like behavior. In the present study, the BaseSpace analysis did not reveal an improvement of aberrant *Wnt* gene expression in the VPA + Vehicle/Control + Vehicle groups vs. VPA + Rapamycin/VPA + Vehicle groups. Further studies are needed to identify the specific pathway that interacts with the mTOR signaling pathway to impair social interaction in VPA-exposed animals. In the present study, we found that rapamycin treatment improved social interactions deficits in VPA-exposed mice in both adolescence and adulthood. Although early treatment is advantageous for improving symptoms of autism in general, our data suggest that rapamycin may be a treatment candidate for ASD patients who are exposed to VPA in utero in both adolescence and adulthood.

Vehicle-treated VPA-exposed mice exhibited the aberrant expression of genes that are associated with the mTOR signaling pathway, and rapamycin treatment did not affect the expression of these genes. Vehicle-treated VPA-exposed mice exhibited an increase in S6 protein phosphorylation compared with vehicle-treated control mice, and rapamycin treatment decreased S6 phosphorylation in VPA-exposed mice. A recent study reported that lymphoblastoid cell lines from patients with idiopathic autism presented an elevation of S6 phosphorylation through an increase in the expression of PI3K catalytic subunit p110δ compared with controls [36]. Rapamycin treatment improved social interaction deficits in *Tsc1*^*+/−*^ and *Tsc2*^*+/−*^ mice and attenuated the protein levels of phosphorylated S6K, which phosphorylates S6 protein, in *Tsc2*^*+/−*^ mice [21]. We speculate that increases in the phosphorylation of S6 are associated with social interaction deficits in animal models of ASD and ASD patients who present an aberrant mTOR signaling pathway.

Meikle et al. (2008) reported that brain levels of rapamycin remained sufficiently high to inhibit mTOR throughout the 48 h period after rapamycin administration (6 mg/kg, i.p.) in mice. Immunoblot analyses of brain lysates that were collected 24 h after rapamycin administration (6 mg/kg, i.p.) showed a reduction of pS6 in *Tsc1* ^null-neuron^ mice [35]. In the present study, we treated mice with 10 mg/kg rapamycin. We collected brains on the same day after the end of the social interaction test in adulthood. The brains were immediately frozen in liquid nitrogen after collection and stored at − 80 °C until Western blot analysis. Thus, we presumed that rapamycin continued to exert its effects in VPA-exposed mice.

The rapamycin-induced improvements in social interaction deficits were transient in VPA-exposed mice, in which we found that the effects of rapamycin treatment disappeared within 5 weeks (see rapamycin-treated VPA-exposed mice in Fig. 2e and vehicle-treated VPA-exposed mice in Fig. 2f). As mentioned above, brain levels of rapamycin remained sufficiently high to inhibit mTOR throughout the 48 h period after rapamycin administration in mice [35]. This previous study indicates that the effects of rapamycin are still evident 2 days after administration. To our knowledge, no studies have evaluated the effects of rapamycin on improving social interaction deficits longer than 2 days after administration. A rat pharmacokinetic study showed that the T_1/2_ of rapamycin was 14.0 h for intravenous administration and 33.4 h for oral administration [39]. In the present study, the effects of rapamycin treatment disappeared within 5 weeks. Further studies are needed to clarify the effects of rapamycin treatment on social interaction deficits by investigating specific treatment periods, doses, timing, combined drugs and therapies, and the duration of the effects.

Valproic acid is a histone deacetylase (HDAC) inhibitor that plays a role in transcriptional regulation [40]. It is used for the treatment of spinal muscular atrophy, in which it increases the survival motor neuron (SMN) protein volume [41]. Valproic acid is associated with the regulation of protein expression. Although VPA does not have 1:1 reactivity with S6, the action of the HDAC inhibitor may have influenced the expression of total S6 in the present study.

Overactivation of the mTOR signaling pathway elicits the pathology of ASD. Previous studies and the present data demonstrate overactivation of the mTOR signaling pathway in ASD. To our knowledge, only one other study has reported a decrease in mTOR signaling in VPA-exposed animals [32], but it investigated rats rather than mice, evaluated different brain regions, and assessed the animals at different ages. Future studies should delineate the specific brain regions that are associated with ASD symptoms, and mTOR signaling pathways can then be investigated in each of these brain regions.

We found two negatively correlated genes, *Fyb* and *A330094K24Rik*, between vehicle-treated VPA-exposed mice vs. vehicle-treated control mice and rapamycin-treated VPA-exposed mice vs. vehicle-treated VPA-exposed mice. Although *A330094K24Rik* is registered with Mouse Genome Informatics (http://www.informatics.jax.org/), there is no detected information for *A330094K24Rik*. *Fyb* is a FYN binding protein that acts as an adapter protein of FYN [42]. Fyb/SLP-associated protein is a negative regulator of interleukin 2 (IL-2) transcription [43]. IL-2 plays an important role in immunoregulatory functions that are related to the central nervous system and is reportedly associated with ASD [44]. *Fyb* is a candidate gene in bioinformatic analyses of ASD [45]. Gera et al. reported that rapamycin treatment decreased the translational ratio of *Fyb* in LAPC-4 cells in which AKT is highly phosphorylated by transfection of myristoylated AKT [46]. In the present study, we found that *Fyb* is in the network correlated with S6K, which is downstream of the mTOR signaling pathway (Fig. 4). *Fyb* in this network is associated with inflammatory cytokine and chemokine production [47]. Cytokines and chemokines play a role in developmental cell death, synapse refinement, and phagocytosis during development [48]. The aberrant expression of cytokines and chemokines is associated with the pathology of ASD [49]. *Fyb* may be a key molecule in ASD with an aberrant mTOR signaling pathway.

Previous studies analyzed associations between VPA-exposed mice and genes that are associated with ASD [50, 51], but the characteristics of gene expression in whole brains from VPA-exposed mice have not yet been comprehensively investigated. The present study is the first to comprehensively investigate gene expression in VPA-exposed mice. The present study found positive correlations between gene expression in VPA-exposed mouse brains and curated studies of Diseases, Pharmaco, and Knockout mice. Upregulated and downregulated genes have shown positive correlations with 148 and 136 diseases, respectively (Additional file 2: Table S2a). These results indicate that each up- or downregulated gene in VPA-exposed mouse brains showed similarities to Diseases other than ASD. Scores for Vitamin D deficiency and Injury of the eye region were greater than 90. Interestingly, vitamin D deficiency was assessed as a risk factor for ASD [52]. A recent clinical study reported that oral vitamin D supplementation in children for ASD improved Childhood Autism Rating Scale scores compared with the placebo group [53]. Moreover, vitamin D acts as an inhibitor of the mTOR signaling pathway. Vitamin D activates DNA-damage-inducible transcript 4 (DDIT4), which facilitates activation of the Tsc1/Tsc2 complex; therefore, vitamin D treatment suppresses downstream mTOR activity [54]. Investigating associations between ASD and the comorbid diseases may be necessary to clarify the pathogenesis of ASD.

Upregulated genes showed a positive correlation with 387 compounds, and downregulated genes showed a positive correlation with 446 compounds. The top compounds with a positive correlation were Glysidol and Ozagrel. Previous studies reported that these compounds were associated with neurogenesis and neurite outgrowth in the developmental mouse brains [55–58]. Although VPA is used to model ASD in animals, unknown is the way in which it causes ASD-like symptoms [59]. Thus, analyzing associations between the compounds that are presented in Additional file 2: Table S2b and ASD-like symptoms may contribute to a better understanding of the pathogenesis of ASD that is induced by VPA exposure.

Alterations of the expression of genes (both upregulated genes and downregulated genes) were similar to PRKG1 KO mice (Additional file 2: Table S2c). PRKG1 KO mice are used as a mouse model of sleep disorders [60]. Interestingly, patients with ASD have a high incidence of sleep disturbances [61]. Additionally, a previous study reported that VPA-exposed rats exhibited a disruption of normal sleep architecture and a reduction of the expression levels of GAD65 and GAD67, which are involved in sleep/wakefulness, in cortical tissue [62]. Investigations of ASD-like symptoms in KO mice and other animal models of ASD will contribute to a better understanding of the pathogenesis of ASD and medication development.

In the present study, we found that rapamycin treatment improved impairments in social interaction in both adolescence and adulthood. The expression of two genes (*Fyb* and *A330094K24Rik*) and S6 phosphorylation were reduced by rapamycin treatment. Altogether, these results suggest that an aberrant mTOR signaling pathway is associated with impairments in social interaction in VPA-exposed mice, and rapamycin may be an effective treatment for adolescent and adult patients with not only particular syndromic ASD but also non-syndromic ASD with an aberrant mTOR signaling pathway.

## Additional Files

Additional file 1: **Table S1.** Gene expression in each group. Gene expression in vehicle-treated VPA-exposed mice / vehicle-treated control mice (a) and rapamycin-treated VPA-exposed mice / vehicle-treated VPA-exposed mice (b). *p* values were produced by *t*-test comparisons between VPA-treated and control sample (rapamycin-treated and non-treated samples) probeset intensity values. Ranks are based on fold changes. Genes are arranged in descending order of fold change. (XLSX 403 kb)

Additional file 2: **Table S2.** Each atlas application score from BaseSpace. Each individual tab represents Disease, Pharmaco, and Knockout atlas application. (a) Disease atlas application score. (b) Pharmaco atlas application score. (c) Knockout atlas application score. From BaseSpace. (XLSX 59 kb)

## Abbreviations

ASD: autism spectrum disorder
Fyb: FYN binding protein
KO: knockout
MAPK: mitogen-activated protein kinase
mTOR: mammalian/mechanistic target of rapamycin
mTORC1: mTOR complex 1
mTORC2: mTOR complex 2
NF1: neuronal fibromatosis 1
NMDA: *N*-methyl-D-aspartate
PI3K: phosphatidylinositol-3 kinase
PIKK: phosphatidylinositol-3 kinase (PI3K)-related kinase
PTEN: tensin homolog deleted on chromosome 10
TSC: tuberous sclerosis complex
VPA: valproic acid
